# Royal Medical and Chirurgical Society

**Published:** 1896-12-12

**Authors:** 


### Medical Progress and Hospital Clinics.

*[The Editor will be glad to receive offers of co-operation and contributions from members of the profession. All letter should be addressed to* The Editor, at the Office, 28 & 29, Southampton Street, Strand, London, W.C.]

### ROYAL MEDICAL AND CHIRURGICAL SOCIETY.

A very curious case related by Dr. Charlton Bastian raised many interesting points both as to cerebral localisation and the extent to which the opposite side of the brain can develop compensatory faculties.

The patient was a right-handed man, who had an attack of right hemiplegia in December, 1877, and had been frequently under observation up to March this year, when he died in consequence of another apoplectic attack, this time due to thrombosis of the right middle cerebral artery. When first seen in 1878 he was found to be incompletely paralysed on the right side, with incomplete hemianæsthesia on the same side. Up to 1883 there were slight convulsive attacks; there was then an interval of twelve years, but during the last year of his life he had three severe fits, each of which was followed by a temporary aggravation of the paralysis of the right side. Within a couple of months of his coming under observation his speech defects assumed the form which they preserved during the next eighteen years with remarkable constancy. His voluntary speech was throughout limited to a very few words; though he could repeat words which he heard, and he understood everything that was said to him. This limitation of speech was judged to depend upon an impaired activity of the auditory word-centre. He spent much of his time in reading, and undoubtedly understood what he read. He could not, however, read a single word aloud, nor could he write a single word either from dictation or spontaneously, though he could copy the same word readily with his left hand when he had it written before him. His inability to read aloud or name at sight, whilst he could understand what he read, and repeat words, was considered to point to the existence of a defect in the visuo-auditory commissure; whilst his inability to write a word from dictation, coupled with his ability to copy the same word, seemed to show a similar defect in the other half of the commissure (the audito-visual) between the auditory and the visual word-centres. In addition to the above-mentioned defects there was also an aphemic difficulty of utterance. The unaltered existence of all these troubles was verified even as late as one month before his death.

On examination of the brain, complete atrophy of the convolutions in the territory supplied by the left middle cerebral artery was found. The atrophy had also extended inwards so as to lay open the lateral ventricle, so that the whole of this region was occupied by a large pseudo-cyst. The supra-marginal and angular gyri, as well as the posterior two-thirds of the upper temporal convolution, were included in the parts which had completely disappeared.

The difficulties in reconciling the persistent and often verified clinical condition with the post-mortem record were extreme. In the absence of word-deafness and word-blindness as initial symptoms, one could not suppose that complete softening at first existed in the auditory and the visual word-centres. Yet the post-mortem showed complete atrophy in these regions, although there had been no appreciable change in the speech defects. It became necessary, then, to suppose that the process of gradual atrophy must have coincided with an equally gradual development in the functional activities of the corresponding convolutional regions of the right hemisphere. What, however, was altogether novel and surprising was that restoration should only have taken place in such a very imperfect manner; that imperceptibly, as it were, the imperfect functioning of the left hemisphere and no more should have been taken on by the right hemisphere.

In discussing the matter, Sir William Broadbent said he did not feel warranted in accepting the view that the changes found in the left cerebral hemisphere had been progressive. He thought that in this case the afferent paths to the highest centres were open. Failure only came on when expression of ideas in words was called for. The whole defect was in the motor portion of the speech-mechanism. The "naming" centre was intact, but there was a destruction of the tracts between the "naming" and the "propositionising" centres. Sir William Broadbent, did not explain how it happened that there was complete destruction of the supra-marginal and angular gyri and the posterior two-thirds of the upper temporal convolution without the occurrence of word-blindness or word-deafness. In fact, he explained what the patient had lost, but not what he had retained.

In the adjourned discussion on December 8th Mr. Spencer Watson drew attention to the fact that the patient had at one time more power of expression as regards numerals than words, and he suggested that this might have some relation to the fact that numerals were learned by rote in childhood, did not present the same psychical conceptions to the mind as words did, and were associated with tactile sensation.

The President related a case which well illustrated the absolute separation which sometimes existed between aphasia and agraphia. As illustrating the same point, he drew attention to the fact that the great lexicographer, Samuel Johnson, once, in his later years, found that he had entirely lost his speech, but wrote Latin verses to prove to himself that he had not lost his wits.

In reply, Dr. Bastian criticised Sir William Broadbent's statements in regard to the existence of a "naming" and "propositionising" centre, which, he maintained, could not be localised as the auditory and visual word-centres could be. He also dwelt on the fact that Sir W. Broadbent did not appear to assume any commissural connection between the auditory and the visual word-centres, nor, in fact, any special word-centre, either visual or auditory.

Considering the large number of neurologists in London, one could not but regret that so well reported a case, and one apparently so opposed to widely accepted dogmas as to cerebral physiology, should not have led to a better discussion.

